# Mutation-induced loss of *APP* function causes GABAergic depletion in recessive familial Alzheimer’s disease: analysis of Osaka mutation-knockin mice

**DOI:** 10.1186/s40478-017-0461-5

**Published:** 2017-07-31

**Authors:** Tomohiro Umeda, Tetsuya Kimura, Kayo Yoshida, Keizo Takao, Yuki Fujita, Shogo Matsuyama, Ayumi Sakai, Minato Yamashita, Yuki Yamashita, Kiyouhisa Ohnishi, Mamiko Suzuki, Hiroshi Takuma, Tsuyoshi Miyakawa, Akihiko Takashima, Takashi Morita, Hiroshi Mori, Takami Tomiyama

**Affiliations:** 1 Department of Translational Neuroscience, Osaka City University Graduate School of Medicine, 1-4-3 Asahimachi, Abeno-ku, Osaka, 545-8585 Japan; 2 Core Research for Evolutional Science and Technology, Japan Science and Technology Agency, Kawaguchi, Japan; 3 Department of Aging Neurobiology, Center for Development of Advanced Medicine for Dementia, National Center for Geriatrics and Gerontology, Obu, Japan; 4 Department of Molecular Genetics, Osaka City University Graduate School of Medicine, Osaka, Japan; 5 Section of Behavior Patterns, Center for Genetic Analysis of Behavior, National Institute for Physiological Sciences, Okazaki, Japan; 6 Biosignal Research Center, Kobe University, Kobe, Japan; 7 Department of Clinical Neuroscience, Osaka City University Medical School, Osaka, Japan; 8 Present address: Life Science Research Center, University of Toyama, Toyama, Japan

**Keywords:** Alzheimer’s disease, Recessive mutation, Knockin mouse, Loss of function, GABA

## Abstract

The E693Δ (Osaka) mutation in *APP* is linked to familial Alzheimer’s disease. While this mutation accelerates amyloid β (Aβ) oligomerization, only patient homozygotes suffer from dementia, implying that this mutation is recessive and causes loss-of-function of amyloid precursor protein (APP). To investigate the recessive trait, we generated a new mouse model by knocking-in the Osaka mutation into endogenous mouse *APP*. The produced homozygous, heterozygous, and non-knockin littermates were compared for memory, neuropathology, and synaptic plasticity. Homozygotes showed memory impairment at 4 months, whereas heterozygotes did not, even at 8 months. Immunohistochemical and biochemical analyses revealed that only homozygotes displayed intraneuronal accumulation of Aβ oligomers at 8 months, followed by abnormal tau phosphorylation, synapse loss, glial activation, and neuron loss. These pathologies were not observed at younger ages, suggesting that a certain mechanism other than Aβ accumulation underlies the memory disturbance at 4 months. For the electrophysiology studies at 4 months, high-frequency stimulation evoked long-term potentiation in all mice in the presence of picrotoxin, but in the absence of picrotoxin, such potentiation was observed only in homozygotes, suggesting their GABAergic deficit. In support of this, the levels of GABA-related proteins and the number of dentate GABAergic interneurons were decreased in 4-month-old homozygotes. Since APP has been shown to play a role in dentate GABAergic synapse formation, the observed GABAergic depletion is likely associated with an impairment of the APP function presumably caused by the Osaka mutation. Oral administration of diazepam to homozygotes from 6 months improved memory at 8 months, and furthermore, prevented Aβ oligomer accumulation, indicating that GABAergic deficiency is a cause of memory impairment and also a driving force of Aβ accumulation. Our findings suggest that the Osaka mutation causes loss of APP function, leading to GABAergic depletion and memory disorder when wild-type APP is absent, providing a mechanism of the recessive heredity.

## Introduction

Cerebral accumulation of Aβ oligomers is believed to be the initial step in the pathogenesis of Alzheimer’s disease (AD) [2, 29]. Aβ is generated from amyloid precursor protein (APP) by the function of two distinct enzymes, β- and γ-secretase [14]. γ-Secretase is a complex composed of at least four membrane proteins in which presenilin 1 or presenilin 2 constitutes the catalytic subunits. Genetic studies have found that mutations in *APP* (chromosome 21), *PSEN1* (chromosome 14), and *PSEN2* (chromosome 1) are linked to familial AD [3].

The inheritance of pathogenic mutations can be defined into two types, dominant and recessive, according to the impact of the mutant allele on the phenotype [22]. Dominant mutations cause disease even in heterozygotes by 1) gain-of-toxic-function of the gene product, 2) loss-of-function with dominant-negative effect, and 3) loss-of-function if 50% level of the normal gene product is not sufficient for normal gene function (haploinsufficiency). On the other hand, recessive mutations cause disease only in homozygotes primarily by loss-of-function: heterozygotes do not show pathogenic phenotypes, since the wild-type counterpart overcomes the deficiency of the mutant protein.

All pathogenic mutations in *APP*, *PSEN1*, and *PSEN2* affect Aβ production and/or aggregation and most of them are dominant [3]. Meanwhile, there are few recessive mutations reported. The E693Δ (Osaka) mutation in APP, which corresponds to E22Δ in Aβ, is the first recessive mutation identified in AD [25]. So far, two pedigrees with this mutation have been identified in Japan: one is in Osaka [20, 25] and the other is in the Inland Sea of Japan [11]. In both pedigrees, only homozygotes (2 members in Osaka and 3 members in the latter) suffer from dementia. However, it is unclear what kind of loss-of-function is induced in patients. Studies with synthetic peptides revealed that this mutation accelerates Aβ oligomerization, but never causes Aβ fibrillization. When injected into the cerebral ventricle of normal rats, the mutant Aβ peptides inhibited long-term potentiation (LTP) more potently than wild-type peptides [25]. Furthermore, in APP transgenic mice harboring this mutation (referred to as APP_OSK_ mice), the produced Aβ formed abundant oligomers and accumulated within neurons to cause synaptic and cognitive impairment without forming amyloid plaques [26]. The enhanced Aβ oligomer formation and the lack of senile plaques have also been suggested in homozygous human patients, which were surmised from Western blot of CSF samples and brain amyloid imaging [11, 20, 25]. Such phenotypes appear to represent gain-of-toxic-function, but nevertheless they are seen only in homozygotes. The second recessive mutation is the A673V mutation in APP, which corresponds to A2V in Aβ [5]. This mutation has been shown to increase Aβ production and accelerate Aβ fibrillization, but the mutant Aβ do not aggregate when co-incubated with wild-type Aβ. Furthermore, what kind of loss-of-function is induced by this mutation is also unclear. Interestingly, A673T mutation at the same position in APP shows protective effects against AD by reducing Aβ production and aggregation [7].

To investigate the genetic traits of recessive AD mutations more closely, we generated a new mouse model by knocking-in the Osaka mutation into endogenous mouse *APP*. The produced knockin mice (referred to as OSK-KI mice) displayed Aβ pathologies only in homozygotes. We noticed that their memory impairment preceded Aβ accumulation and accompanied GABAergic depletion, which was presumably caused by the loss-of-function of APP. Thus, the present study provides new insights into the mechanism underlying the recessive heredity of the Osaka mutation.

## Materials and methods

### Generation of OSK-KI mice

Mice harboring the Osaka mutation in their Aβ sequence were generated by knocking-in this mutation into endogenous mouse *APP* by homologous recombination in embryonic stem cells. Mouse *APP* contains 18 exons, and Aβ is coded in exons 16 and 17 (GenBank: U82624.1). The targeting vector (pTVneo/*APP*) was constructed according to the method of Thuy le et al. [24]. Three DNA fragments (5′, middle, and 3′) were produced by PCR from 129Sv mouse genomic DNA using the primer pairs indicated in Table 1 followed by restriction enzyme cleavage. The 5′ PCR fragment (4.4 kb) contained *APP* intron 15, exon 16, intron 16, and the 5′ region of exon 17. The reverse PCR primer used for this fragment was designed to have a deletion of codon693 (GAA) in exon 17 (i.e. the Osaka mutation). The middle PCR fragment (0.6 kb) contained the 3′ region of exon 17 and 5′ region of intron 17. The two DNA fragments were ligated and used as the 5′ arm. The 3′ PCR fragment (5.1 kb) containing intron 17 was used as the 3′ arm. The neomycin-resistance gene, driven by the phosphoglycerate kinase 1 promoter, with flanking lox-P sequences was inserted into the arms. Mouse embryonic stem cells (1 × 10^7^ cells/mL) were transfected with the linearized targeting vector (20 μg) by electroporation and cultured in selection medium containing 150 μg/mL geneticine (G418). Of 200 neomycin-resistant clones, only one (0.5%) was a homologous recombinant, which was determined by Southern blot hybridization using the 5′ and 3′ probes (data not shown). The clone was aggregated with C57BL/6-DBA2 F1 mouse morulae, and the chimera embryos were transplanted into pseudopregnant mice. The produced chimeric male mice were mated with C57BL/6 J females to obtain germline transmitting KI mice that were backcrossed to the C57BL/6 J background for more than ten generations. Homozygous KI mice were generated by crossing heterozygotes. Genotyping was performed by PCR from mouse tail DNA using the primers indicated in Table 1. All animal experiments were approved by the committee of Osaka City University and were performed in accordance with the Guide for Animal Experimentation, Osaka City University. Every effort was made to minimize the number of animals used and their suffering.

**Table 1:** PCR primers used for targeting vector construction, probe preparation, and mouse genotyping

Name	Sequence
Targeting vector	
TV-Am-5'-F (NotI)	5'-ATAAGAATGCGGCCGCGTAGGAAGGCCCAGCTAGAAGGAAATGGG-3'
TV-Am-5'-R (NarI)	5'-CCGATGATGGCGCCTTTGTTCGAACCCACATC (ΔTTC) AGCAAAGAACACCTTCGAAAGGAAGCCG-3'
TV-Am-M-F (NarI)	5'-CGGCTTCCTTTCGAAGGTGTTCTTTGCT-3'
TV-Am-M-R (AscI)	5'-TTGGCGCGCCAGTTAACTAGGCCTAATGTTCCTCCATGGTAACCACGC-3'
TV-Am-3'-F (PmeI)	5'-AGCTTTGTTTAAACAGGCTGTTGCCCTGAACTTCCACCTGAG-3'
TV-Am-3' R (AatII)	5'-GGGGTTAGACGTCCCATTGGGTGTGACCCCACTTCAGAG-3'
Southern probes	
Am-5'-probe F	5'-TCCCCCACCCCCTGTTATAAAAGG −3'
Am-5' probe R	5'-TGCTCTTTAAATCACCCCGGTTGC-3'
Am-3'-probe F	5'-TCCTCTCGTCTTCCAACGCGGCTT −3'
Am-3'-probe R	5'-CCGCCAGGCCAGAGCTCTACAGCA-3'
Genotyping	
KI/WT forward	5'-CCTAGGGACCCACCAACTCACGCT-3'
WT reverse	5'-GGTGGAAGTTCAGGGCAACAGCCT-3'
KI reverse	5'- TCTCCTGTCATCTCACCTTGCT-3'

The targeting vector was constructed with three DNA fragments (5', middle, and 3') derived from mouse APP gene. The three fragments were produced by PCR using the primer pairs of TV-Am-5'-F/R, TV-Am-M-F/R, and TV-Am-3'-F/R, respectively. Underlines indicate restriction sites. ΔTTC represents a deletion of codon693 (GAA). Southern blot hybridization was carried out with 5' and 3' probes that were prepared by PCR using the primer pairs of Am-5' probe F/R and Am-3' probe F/R, respectively. Genotyping of mice was performed by PCR using the three primer mixtures. The KI reverse primer was in the neomycin-resistance gene. Wild-type allele produced a 780 bp PCR product with the primer pair of KI/WT forward and WT reverse, while KI allele showed an 1820 bp PCR product with the primer pair of KI/WT forward and KI reverse

### Antibodies

Mouse monoclonal antibodies to Aβ oligomers (11A1; IBL, Fujioka, Japan), the presynaptic marker synaptophysin (SVP-38; Sigma, St. Louis, MO), the astrocyte marker glial fibrillary acidic protein (GFAP) (Cappel, Aurora, OH), the mature neuron marker NeuN (Chemicon, Temecula, CA), and the GABAergic neuron marker parvalbumin (PARV-19; Sigma) were purchased. Rabbit polyclonal antibodies to the microglia marker Iba-1 (Wako, Osaka, Japan), GABA-synthetic enzyme glutamate decarboxylase (GAD) 65/67 (EMD Millipore, Temecula, CA), vesicular GABA transporter (VGAT) (Proteintech, Rosemont, IL), vesicular glutamate transporter (VGLUT) 1/2 (Abcam, Cambridge, MA) and actin (Sigma) were also purchased. Mouse monoclonal antibody to pSer396/Ser404-tau (PHF-1) was a kind gift from Dr. Peter Davies (Department of Pathology, Albert Einstein College of Medicine, Bronx, NY), and rabbit polyclonal antibodies to Aβ42 (Ter42), Aβ N-terminus (β001), and APP C-terminus (C40) were prepared in our laboratory.

### Behavioral analysis

Spatial reference memory of male mice was assessed in Osaka City University at 4, 6, and 8 months using the Morris water maze, as described previously [28]. In addition, comprehensive behavioral test battery was performed in the National Institute for Physiological Sciences on 8-month-old male mice to study their sensorimotor functions, locomotor activity, social behavior, anxiety-like behavior, depression-like behavior, and learning/memory, as described previously [10].

### Immunohistochemical analysis

Brain sections were prepared as described previously [26]. Aβ accumulation (Ter42, β001 and 11A1), abnormal tau phosphorylation (PHF-1), synapse loss (synaptophysin), and glial activation (GFAP and Iba-1), were examined as described previously [26], where only for Aβ staining, sections were pretreated by boiling in 0.01 N HCl (pH 2) for 10 min to expose epitopes. Neuronal loss was assessed with anti-NeuN antibody with (entorhinal) or without (hippocampus) boiling sections in 10 mM citrate buffer (pH 6) for 30 min. GABAergic interneurons were stained with anti-parvalbumin antibody after sections were boiled in 10 mM citrate buffer (pH 6) for 30 min.

### Biochemical analysis

To determine the expression levels of APP, brain tissues were homogenized in 5 volumes of 50 mM Tris–HCl (pH 7.6), 150 mM NaCl (Tris-buffered saline, TBS) containing 1% Triton X-100 and protease inhibitor cocktail (P8340; Sigma). After agitation at 4 °C for 1 h, the homogenates were centrifuged at 1000 x g for 15 min at 4 °C to remove insoluble materials. The supernatants were subjected to Western blot with antibodies to APP C-terminus (C40) and actin. In different experiments, hippocampal tissues were dissected from mouse brains and homogenized in 4 volumes of TBS containing P8340. The levels of synaptophysin, GAD65/67, VGAT, VGLUT1/2, and actin were examined by Western blot with corresponding antibodies. Signals were visualized and quantified using an ImageQuant LAS 500 (GE Healthcare Bio-Sciences, Uppsala, Sweden). The remaining brain tissues, not including the cerebellum, were also homogenized in 4 volumes of TBS containing P8340 and separated into TBS-soluble and SDS-soluble fractions by 2-step ultracentrifugation, the latter of which were dialyzed against TBS, essentially as described previously [28]. The levels of Aβ42 in each fraction were measured using the Sensolyte anti-mouse/rat β-amyloid (1–42) quantitative ELISA kit Colorimetric (Anaspec, Fremont, CA). Aβ oligomers and phosphorylated tau in the homogenates were measured by direct ELISA with 11A1 antibody and by using the Human Tau [pS396] ELISA kit (Invitrogen, Camarillo, CA), respectively, as described previously [28].

### Electrophysiological analysis

Synaptic plasticity was examined by electrophysiology using hippocampal slices, essentially as described previously [32]. Transverse hippocampal slices (350 μm thick) were prepared in ice-cold artificial cerebrospinal fluid (aCSF; NaCl 124 mM, KCl 3 mM, NaHCO_3_ 26 mM, NaH_2_PO_4_ 1.25 mM, CaCl_2_ 2 mM, MgSO_4_ 1 mM, and D-glucose 10 mM) containing 1 mM kynurenic acid. Slices were allowed to recover in aCSF at room temperature for 1–2 h and then transferred to the recording chamber, in which they were perfused at a rate of 2 ml/min with aCSF at 32 °C. Electrical stimulation was applied onto the molecular layer of dentate gyrus using a bipolar tungsten electrode, and field excitatory postsynaptic potential (fEPSP) was recorded using a glass electrode in the same region at 200-μm distance from the stimulating electrode. Baseline stimulation was 15- to 20-μA constant current pulse, which induces fEPSP at a level 50% the maximum amplitude, 100-μsec pulse duration, and 30-s pulse interval. After baseline recording for 15 min, high-frequency stimulation (HFS; 3 trains of 100 Hz, 100 pulses, 120-s train interval) with an intensity 2-fold higher than that of baseline stimulation was delivered. The produced fEPSP was recorded for 60 min in the presence or absence of a GABA_A_ receptor antagonist picrotoxin (Sigma) at 40 μM. fEPSP slopes were compared at 60 min.

### Diazepam treatment to OSK-KI mice

Diazepam (Sigma), a positive allosteric modulator of GABA_A_ receptor, was dissolved to 10 μg/ml in 0.5% low-viscosity carboxymethylcellulose (CMC; Sigma). Diazepam is usually prescribed to adult humans at 2 to 10 mg orally 2 to 4 times a day for anxiety and seizures (https://www.drugs.com/dosage/diazepam.html). Thus, its minimum daily dose for humans is 4 mg. Assuming that mean body weights of adult humans and mice are 60 kg and 30 g, respectively, the minimum daily dose for mice corresponds to 2 μg. Thus, 200 μl of diazepam (i.e. 2 μg) or CMC solution was orally administered using feeding needles to 6-month-old male homozygotes (*n* = 9–10 per group) 5 days a week (Monday through Friday) for 2 months. Age-matched male non-KI littermates (*n* = 10) administered CMC solution were used as controls. Spatial reference memory was examined at 8 months using the Morris water maze as described above. Daily oral administration of diazepam was continued during the behavioral test. After the behavioral tests, brain sections were prepared and Aβ oligomer accumulation (11A1), synapse loss (synaptophysin), and GABAergic neurons (parvalbumin) were examined by immunohistochemistry as described above.

### Transfection and western blot of mouse Aβ oligomers

Human APP695 constructs with the Swedish (K670 N/M671 L, SW) and Osaka mutations were prepared as described previously [13], from which mouse APP695 constructs were produced by site-directed mutagenesis. HEK293 cells were transfected with human or mouse APP_SW_ and APP_SW/OSK_ constructs, as described previously [13]. The Swedish mutation was introduced just to increase total Aβ production. Three days after transfection, the cells from 5 culture dishes (10 cm diameter) were combined into 1 tube and homogenized by sonication in 1 mL of 1% Triton X-100/TBS containing P8340. After agitation at 4 °C for 1 h, the cell homogenates were centrifuged at 1000 x g for 15 min at 4 °C to remove cell debris. Aliquots of the supernatants were subjected to Western blot to measure APP expression (C40) and actin. Aβ in the remaining supernatants were immunoprecipitated using anti-Aβ antibody β001 and subjected to Western blot with the same antibody, essentially as described previously [13]. Signals were visualized and quantified using an ImageQuant LAS 500.

### Statistical analysis

Comparisons of means between more than two groups were performed using the Bonferroni/Dunn test in immunohistochemical and biochemical analyses, and the comparison of fEPSP slopes at 60 min in electrophysiology was done using the Tukey-Kramer test. Data in behavioral tests were analyzed using ANOVA or repeated measures ANOVA followed by the Tukey-Kramer test. Differences with a *p* value of less than 0.05 were considered significant.

## Results

### Generation of OSK-KI mice

**Fig. 1:**
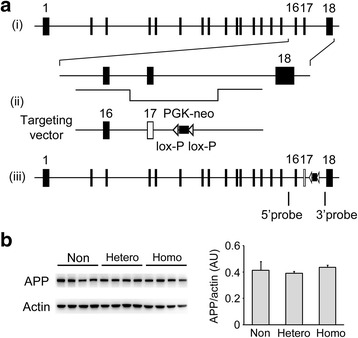
Generation of OSK-KI mice. (**a**) Mice were generated by knocking-in the Osaka mutation (deletion of codon 693) into endogenous mouse *APP* by homologous recombination in embryonic stem cells. (*i*) Mouse *APP* contains 18 exons (black boxes), and Aβ is coded in exons 16 and 17. (*ii*) The targeting vector contains mouse *APP* exon 16, mutant exon 17 with the deletion (white box), and the neomycin-resistance gene driven by the phosphoglycerate kinase 1 promoter (PGK-neo). (*iii*) Homologous recombinants were determined by Southern blotting using the 5' and 3' probes. (**b**) Expression levels of APP in homozygous, heterozygous, and non-KI mice. Brain homogenates at 24 months were subjected to Western blot with antibodies to APP C-terminus (C40) and actin. Each bar represents the mean ± SEM (*n* = 4 for each group). AU, arbitrary unit

OSK-KI mice were generated by homologous recombination with a targeting vector containing mouse *APP* fragment around exon 17 in which codon 693 was deleted (Fig. 1a). The obtained heterozygous male and female KI mice were crossed with each other to produce homozygotes. The ratio of homozygous, heterozygous, and non-KI offspring was approximately 1:2:1. These mice all appeared normal. Homozygotes produced only mutant mouse Aβ, while heterozygotes produced both wild-type and mutant mouse Aβ. There were no differences in the levels of endogenous APP among the homozygote, heterozygote, and non-KI groups (Fig. 1b).

### Memory impairment in OSK-KI mice

**Fig. 2:**
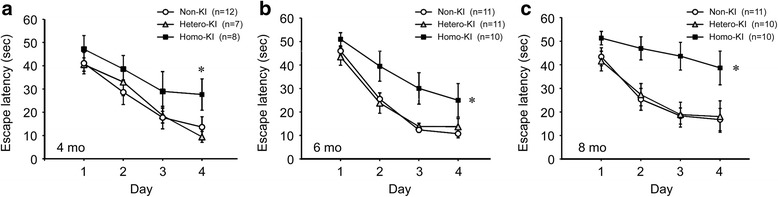
Memory impairment in OSK-KI mice. Spatial reference memory of mice was examined at 4 (**a**), 6 (**b**), and 8 months (**c**) using the Morris water maze. Each point represents the mean latency of five trials per day ± SEM. (**a**) *p < 0.05 versus hetero-KI when means of day 4 were separately compared using the Tukey-Kramer test. (**b**) *p < 0.05 versus non-KI and <0.05 versus hetero-KI. (**c**) *p < 0.05 versus non-KI and <0.05 versus hetero-KI

We initially tested spatial reference memory of OSK-KI mice at 4, 6, and 8 months using the Morris water maze. Compared with non-KI littermates, homozygotes showed impaired memory early at 4 months, whereas heterozygotes maintained memory at similar levels even at 8 months (Fig. 2). This result is in agreement with our previous finding in humans that the Osaka mutation causes dementia in a recessive hereditary manner [25].

Comprehensive behavioral study was performed on 8-month-old OSK-KI mice. Almost no significant differences were observed among homozygotes, heterozygotes, and non-KI mice except for few parameters including body weight, motor coordination, and locomotor activity in dark (Table 2).

**Table 2:** Phenotypes of OSK-KI mice in comprehensive behavioral test battery

Tests	Hetero-KI	Homo-KI	
	(vs. Non-KI)	(vs. Non-KI)	(vs. Hetero-KI)
Somatic parameters			
Body weight	─	─	↓^1^
Body temperature	─	─	─
Grip strength	─	─	─
Wire-hanging time	─	─	─
Light/dark transition test			
Anxiety	─	─	↓^2^
Open field			
Exploratory locomotion	─	─	─
Elevated plus maze			
Anxiety	─	─	─
Rotarod			
Motor coordination	↓^3^	↓^3^	─
Hot plate			
Pain sensitivity	─	─	─
Social interaction test (novel environment)	─	─	─
Social interaction test (Crawley’s version)	─	─	─
Startle Response/Prepulse inhibition			
Sensorimotor gating	↓^4^	─	↑^4^
Porsolt forced swim			
Immobility time (behavioral despair)	─	─	─
Barns maze			
Spatial memory	─	↑^5^	─
Eight-arm radial maze			
Working memory	─	↑^6^	─
T-maze forced alternation			
Working memory	─	─	─
Gait analysis			
Gait ataxia	─	─	─
Fear conditioning			
Contextual fear memory	─	─	─
Tail suspension			
Immobility time (behavioral despair)	─	─	─
Social interaction test (24-h homecage monitoring)	─	↓^7^	─

Comprehensive behavioral test battery was performed on 8-month-old male mice as described previously [10]. The numbers of mice used in each test were *n* = 15–17 for non-KI, *n* = 16–20 for hetero-KI, and *n* = 12–16 for homo-KI except for the social interaction tests (novel environment and 24-h homecage monitoring) in which *n* = 7–9 for non-KI, *n* = 8–10 for hetero-KI, and *n* = 6–8 for homo-KI. ─, no significant difference was detected. ↓^1^, body weight of homo-KI was slightly but significantly (*p* < 0.05) lower than that of hetero-KI. ↓^2^, distance traveled in dark of homo-KI was slightly but significantly (*p* < 0.05) lower than that of hetero-KI. ↓^3^, latency to fall of hetero-KI was significantly (*p* < 0.05) lower than that of non-KI; latency to fall of homo-KI was also lower than that of non-KI, but the difference was not significant. ↓^4^ and ↑^4^, prepulse inhibition of hetero-KI was lower than those of non-KI and homo-KI, but the differences were not significant. ↑^5^, error to 1st of homo-KI was slightly but significantly (*p* < 0.05) lower (i.e. improved) than that of non-KI. ↑^6^, different arm choices in first 8 entries of homo-KI was slightly but significantly (*p* < 0.05) higher (i.e. improved) than that of non-KI. ↓^7^, activity level in dark of homo-KI was significantly (*p* < 0.05) lower than that of non-KI

### Aβ-related neuropathology in OSK-KI mice

**Fig. 3:**
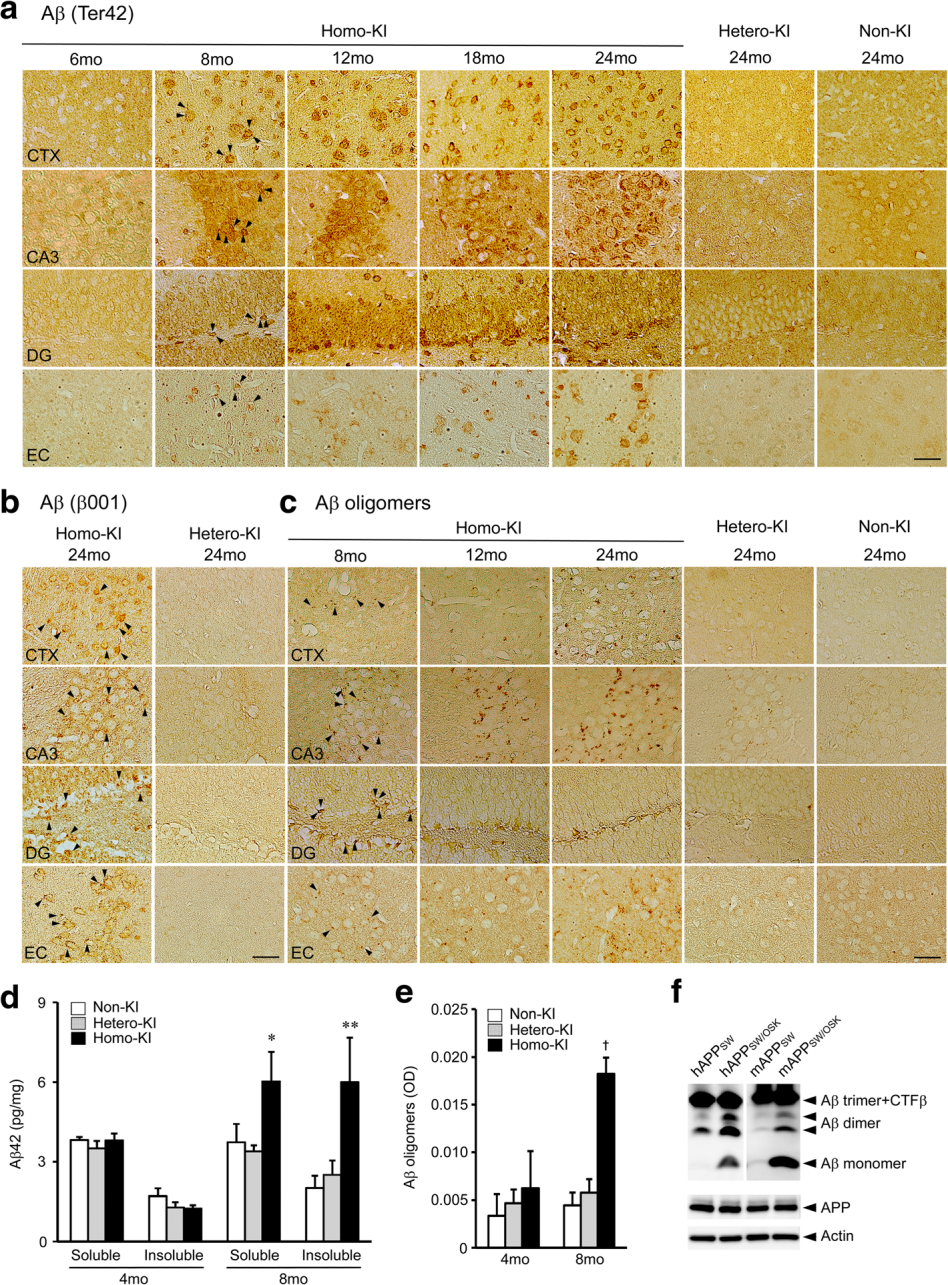
Aβ accumulation in OSK-KI mice. Brain sections were stained with anti-Aβ42 Ter42 (**a**), anti-Aβ N-terminus β001 (**b**) and Aβ oligomer-specific 11A1 antibodies (**c**). Photographs were taken from the posterior parietal association area (PPtA) of the cerebral cortex (CTX), hippocampal CA3 region (CA3), dentate gyrus (DG), and entorhinal cortex (EC). Arrowheads indicate Aβ accumulated within neurons. Scale bar = 30 μm. (**d**) Brain homogenates at 4 and 8 months were separated into TBS-soluble and insoluble (SDS-soluble) fractions and subjected to Aβ42 sandwich ELISA. Each bar represents the mean ± SEM (*n* = 4 for each group). *p = 0.0391 versus hetero-KI, **p = 0.0255 versus non-KI and = 0.0441 versus hetero-KI. (**e**) For Aβ oligomers, brain homogenates were subjected to direct ELISA with 11A1 antibody. Each bar represents the mean ± SEM (*n* = 4 for each group). †*p* = 0.0001 versus non-KI and = 0.0002 versus hetero-KI. (**f**) HEK293 cells were transfected with *human (h)* or *mouse (m)* APP_SW_ and APP_SW/OSK_ constructs. Three days after transfection, the cells were homogenized and subjected to Western blot to measure APP expression (C40) and actin. Intracellular Aβ were immunoprecipitated using anti-Aβ antibody β001 and subjected to Western blot with the same antibody

Then we examined the neuropathology of OSK-KI mice at various ages. Aβ accumulation was visualized by immunohistochemistry with anti-Aβ42 (Ter42), anti-Aβ N-terminus (β001), and Aβ oligomer-specific antibodies (11A1). Homozygotes exhibited intraneuronal accumulation of Aβ in the cerebral cortex, hippocampus, dentate gyrus, and entorhinal cortex at 8 months (Fig. 3a, b). These Aβ were also positive to 11A1 antibody (Fig. 3c), indicating that they formed oligomers. In contrast, heterozygotes showed Aβ accumulation only slightly at 24 months, which was similar to that of age-matched non-KI littermates. None of the three groups displayed extracellular amyloid deposits. An accumulation of Aβ42 and Aβ oligomers in 8-month-old homozygotes was confirmed by ELISA (Fig. 3d, e). The enhanced oligomerization of Osaka-mutant mouse Aβ was also shown in transfected cells. Immunoprecipitation/Western blot analysis revealed that Osaka-mutant mouse Aβ formed oligomers (primarily dimers) more abundantly than wild-type mouse Aβ and accumulated within cells, similarly to Osaka-mutant human Aβ [13] (Fig. 3f). Thus, the Osaka mutation was shown to have Aβ gain-of-toxic-function by which Aβ oligomerization is accelerated, not only in the human but also mouse Aβ sequence. However, this phenotype was seen only in homozygotes, suggesting that the gain-of-toxic-function is insufficient or regulated by some factor.

**Fig. 4:**
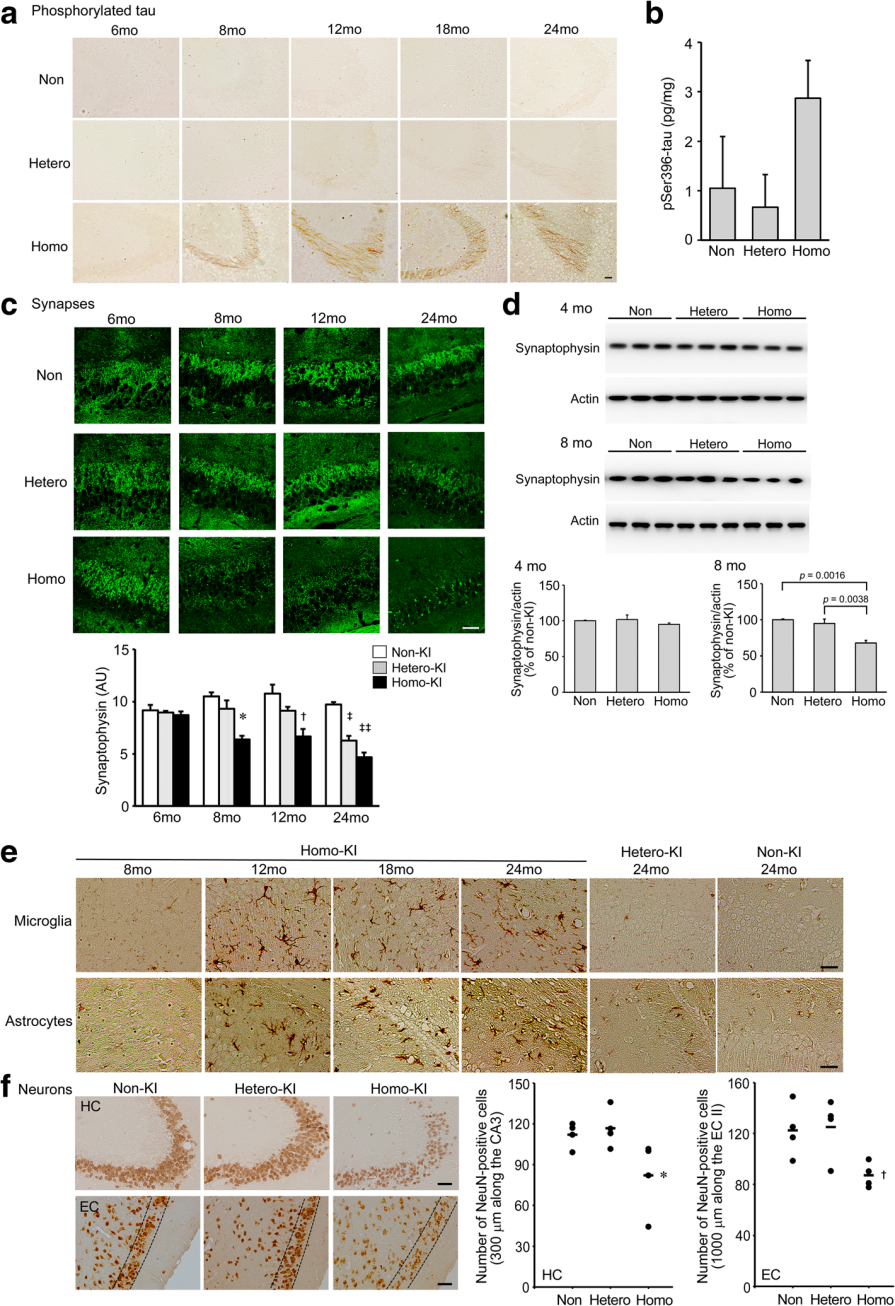
Aβ-related neuropathology in OSK-KI mice. Abnormal tau phosphorylation, synapse loss, glial activation, and neuron loss were examined. (**a**) Brain sections were stained with PHF-1 antibody specific to pSer396/Ser404-tau. Photographs were taken from the hippocampal CA2/3 region. Scale bar = 30 μm. (**b**) Brain homogenates at 8 months were subjected to pSer396-tau sandwich ELISA. Each bar represents the mean ± SEM (*n* = 4 for each group). (**c**) Brain sections were stained with anti-synaptophysin antibody. Photographs were taken from the hippocampal CA3 region. Scale bar = 30 μm. Fluorescence intensities in a constant area were quantified as described previously [28]. Each bar represents the mean ± SEM (*n* = 3 for each group). AU, arbitrary unit. *p = 0.0019 versus non-KI and = 0.0093 versus hetero-KI, †*p* = 0.0052 versus non-KI and = 0.0424 versus hetero-KI, ‡*p* < 0.0001 versus non-KI and = 0.0278 versus homo-KI, ‡‡*p* = 0.0008 versus non-KI. (**d**) Hippocampal homogenates at 4 and 8 months were subjected to Western blot with antibodies to synaptophysin and actin. The signal densities were quantified. Each bar represents the mean ± SEM (*n* = 3 for each group). (**e**) Brain sections were stained with antibodies to Iba-1 (microglia) and GFAP (astrocytes). Photographs were taken from the hippocampus. Scale bar = 30 μm. (**f**) Brain sections at 24 months were stained with anti-NeuN antibody. Photographs were taken from the hippocampal CA2/3 region (HC) and entorhinal cortex (EC). Scale bar = 30 μm. Neu-N-positive neurons in an area within 300 μm along the pyramidal cell layer of the hippocampal CA3 region and in an area within 1000 μm along the layer II (the region between the two broken lines) of the entorhinal cortex were counted, essentially as described previously [26]. *p = 0.0448 versus non-KI and = 0.0245 versus hetero-KI, †*p* = 0.0285 versus non-KI and = 0.0207 versus hetero-KI (*n* = 4 for each group)

Abnormal tau phosphorylation was examined by immunohistochemistry with PHF-1 antibody. Again, only homozygotes showed positive staining in hippocampal mossy fibers at 8 months (Fig. 4a). An increase of phosphorylated tau in 8-month-old homozygotes was confirmed by ELISA (Fig. 4b). Synapse loss was evaluated in the hippocampus by immunohistochemistry with anti-synaptophysin antibody. Compared with non-KI littermates, homozygotes showed a marked decrease in synaptophysin level at 8 months, while heterozygotes exhibited a significant decrease only at 24 months (Fig. 4c). A decrease of synaptophysin in 8-month-old homozygotes was confirmed by Western blot (Fig. 4d). Glial activation was assessed by immunohistochemistry with antibodies to markers of microglia (Iba-1) and astrocyte (GFAP). We observed increased levels in Iba-1-positive and GFAP-positive cells in the hippocampus at 12 months in homozygotes (Fig. 4e). In contrast, no apparent increase was detected in either heterozygotes or non-KI littermates even at 24 months. Finally, neuronal loss was estimated in the hippocampus and entorhinal cortex by immunohistochemistry with an antibody to a mature neuron marker, NeuN. Compared with non-KI littermates, homozygotes but not heterozygotes showed a significant decrease in NeuN-positive cells in both regions at 24 months (Fig. 4f).

These results indicate that the Osaka mutation causes Aβ-related neuropathology in a recessive hereditary manner. However, these phenotypes were recognized only from 8 months, suggesting that a certain unidentified mechanism other than Aβ accumulation underlies the memory disturbance observed in 4-month-old homozygotes.

### Aberrant synaptic activity in OSK-KI mice

**Fig. 5:**
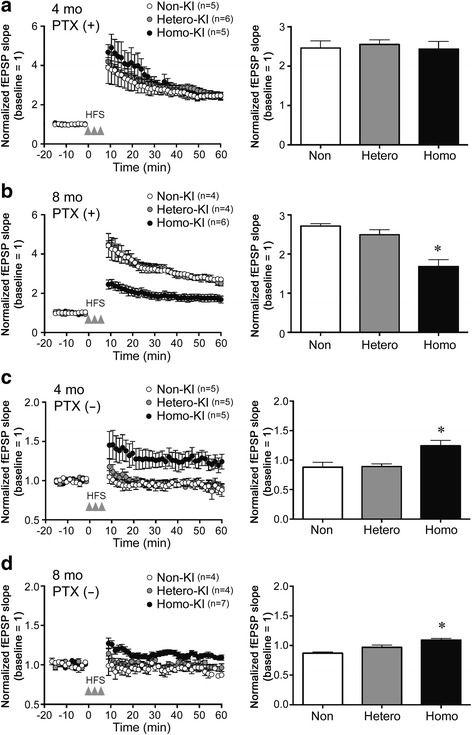
Aberrant synaptic activity in OSK-KI mice. Synaptic plasticity was examined by electrophysiology using hippocampal slices at 4 (**a**, **c**) and 8 months (**b**, **d**). HFS (100 Hz, 100 pulses) was delivered to the dentate gyrus and fEPSP was recorded for 60 min in the same region in the presence (**a**, **b**) and absence (**c**, **d**) of 40 μM picrotoxin. Right panels, the levels of fEPSP slope at 60 min were compared. Each bar represents the mean ± SEM. *p < 0.05 versus non-KI and <0.05 versus hetero-KI

We next studied synaptic plasticity in OSK-KI mice at 4 and 8 months by electrophysiology using hippocampal slices. HFS was delivered to the dentate gyrus, and fEPSP was recorded in the same region in the presence and absence of a GABA_A_ receptor antagonist, picrotoxin, as LTP induction has been shown to be sensitive to GABAergic input [32]. In the presence of picrotoxin, LTP was evoked to similar levels in homozygotes, heterozygotes and non-KI mice at 4 months (Fig. 5a). However, at 8 months, the level of LTP in homozygotes was significantly lower than those of heterozygotes and non-KI mice (Fig. 5b). The LTP inhibition observed in homozygotes was presumably caused by Aβ oligomers, which have been shown to impair glutamatergic signaling [27], a phenomenon similar to that in APP_OSK_ mice [26]. In the absence of picrotoxin, on the other hand, only homozygotes but not heterozygotes nor non-KI mice displayed LTP at 4 and 8 months (Fig. 5c, d). These observations indicate that in heterozygotes and non-KI mice, GABAergic transmission was normal and suppressed LTP induction under the conditions we used. In contrast, the same HFS induced LTP in homozygotes, suggesting that their GABAergic transmission was disrupted. This problem occurred early (4 months) and at the same time as memory impairment. The level of LTP in homozygotes in the absence of picrotoxin was also attenuated at 8 months, probably due to their glutamatergic impairment.

### GABAergic neuron loss in OSK-KI mice

**Fig. 6:**
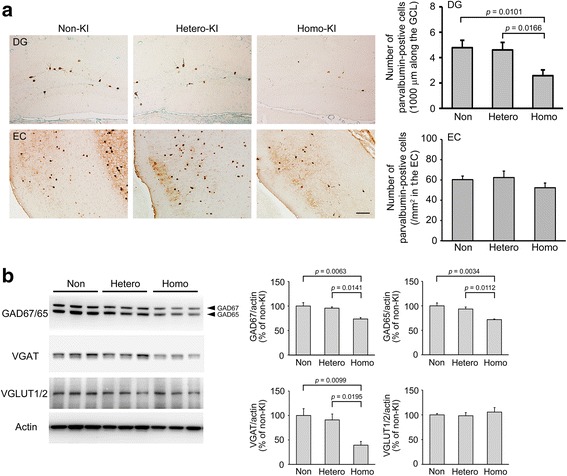
GABAergic neuron loss in OSK-KI mice. (**a**) Brain sections at 4 months were stained with anti-parvalbumin antibody. Photographs were taken from the dentate gyrus (DG) and entorhinal cortex (EC). Scale bar = 30 μm. Parvalbumin-positive GABAergic neurons in the granular cell layer (GCL) of the dentate gyrus within 1000 μm and in an area (700 × 600 μm) of the entorhinal cortex were counted. Each bar represents the mean ± SEM (*n* = 7 for each group). (**b**) Hippocampal homogenates at 4 months were subjected to Western blot with antibodies to GAD67/65 (GABA-synthetic enzyme), VGAT (GABA transporter), VGLUT1/2 (glutamate transporter), and actin. The signal densities were quantified. Each bar represents the mean ± SEM (*n* = 3 for each group)

APP has been reported to be highly expressed in GABAergic interneurons in the dentate gyrus and plays an important role in GABAergic synapse formation [30]. This information led us to speculate that the Osaka mutation may impair the APP function necessary for GABAergic neurons and thereby cause the deficiency of GABAergic transmission in the dentate gyrus. Thus, we measured the number of GABAergic neurons in the dentate gyrus at 4 months. Brain sections were stained with antibody to parvalbumin, a marker of GABAergic neurons. Compared with non-KI littermates, homozygotes showed a significant decrease in parvalbumin-positive cells in the dentate gyrus, but heterozygotes did not (Fig. 6a). In the entorhinal cortex, on the other hand, parvalbumin-positive cells showed a tendency to decrease in homozygotes, but the differences were not significant (Fig. 6a). The decrease of GABAergic neurons in 4-month-old homozygotes was confirmed by Western blot with antibodies to the GABA-synthetic enzyme GAD65/67 and the GABA transporter VGAT (Fig. 6b). In contrast, glutamatergic neurons were not affected in 4-month-old homozygotes, as shown in Western blot with an antibody to the glutamate transporter VGLUT1/2 (Fig. 6b).

### Effects of diazepam treatment on memory and Aβ pathology in OSK-KI mice

**Fig. 7:**
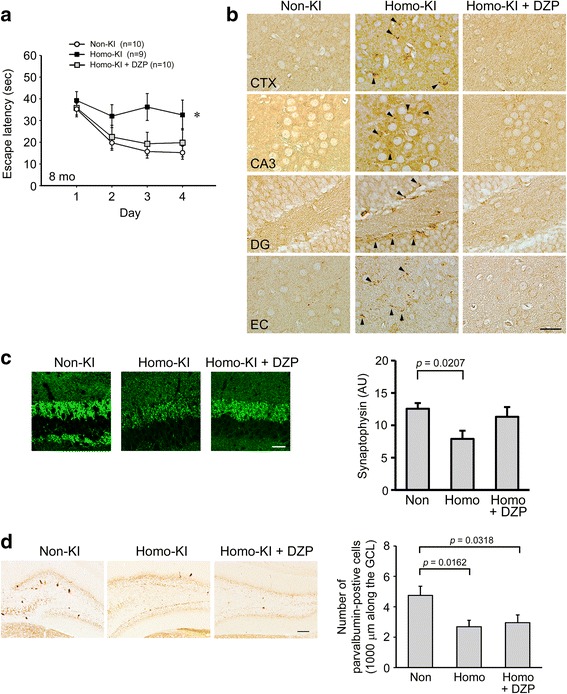
Effects of diazepam treatment on memory and Aβ pathology in OSK-KI mice. Diazepam (DZP) was orally administered to 6-month-old homo-KI mice at 2 μg/day for 2 months. (**a**) Spatial reference memory was examined at 8 months using the Morris water maze. Each point represents the mean latency of five trials per day ± SEM. *p < 0.05 versus non-KI. (**b**) Brain sections were stained with Aβ oligomer-specific 11A1 antibody. Photographs were taken from the PPtA of the cerebral cortex (CTX), hippocampal CA3 region (CA3), dentate gyrus (DG), and entorhinal cortex (EC). Arrowheads indicate Aβ accumulated within neurons. Scale bar = 30 μm. (**c**) Brain sections were stained with anti-synaptophysin antibody. Photographs were taken from the hippocampal CA3 region. Scale bar = 30 μm. Fluorescence intensities in a constant area were quantified. Each bar represents the mean ± SEM (*n* = 5 for each group). AU, arbitrary unit. (**d**) Brain sections were stained with anti-parvalbumin antibody. Photographs were taken from the dentate gyrus. Scale bar = 30 μm. Parvalbumin-positive GABAergic neurons in a constant area in the granular cell layer (GCL) of the dentate gyrus were counted. Each bar represents the mean ± SEM (*n* = 5 for each group)

In homozygotes, GABAergic depletion and memory impairment occurred at 4 months, and Aβ accumulation was detected at 8 months. Is there any causal relationship between these events? Since Aβ production has been shown to depend on neuronal activity [8, 17] and since GABA is an inhibitory neurotransmitter, we speculate that GABAergic depletion may cause aberrant neuronal activation and thereby accelerate Aβ production and accumulation. If so, compensative treatment with GABA agonists would prevent Aβ accumulation in homozygotes. To test this hypothesis, we orally administered diazepam, a member of benzodiazepines and a positive allosteric modulator of GABA_A_ receptor, to homozygotes from 6 months and examined their memory and Aβ pathology at 8 months. Diazepam treatment improved memory (Fig. 7a) and prevented Aβ oligomer accumulation (Fig. 7b) and synapse loss (Fig. 7c), but did not affect parvalbumin-positive GABAergic neurons in the dentate gyrus (Fig. 7d) in homozygotes. These results indicate that Aβ accumulation in OSK-KI mice depends on early GABAergic depletion.

## Discussion

In the present study, we generated a new mouse model of AD by knocking-in the Osaka mutation into endogenous mouse *APP*. The produced OSK-KI mice successfully displayed memory impairment, Aβ oligomer accumulation, and subsequent Aβ-related pathology. Since the exact neuropathology in human patients with the Osaka mutation is not known, we cannot validate the phenotypes of OSK-KI mice at the moment. Nevertheless, it is important that the above phenotypes were seen only in homozygotes, reflecting the recessive heredity of the Osaka mutation that was originally observed in humans [11, 20, 25]. In general, recessive mutations cause disease primarily by loss-of-function of the gene product [22]. The fact that the Osaka mutation is recessive implies that this mutation induces a loss-of-function of APP. Then, what kind of loss-of-function is induced by the Osaka mutation? A hint was found in the paper of Wang et al. [30], where they demonstrated using APP knockout mice that APP is highly expressed in GABAergic interneurons in the dentate gyrus and plays an important role in GABAergic synapse formation. This information led us to speculate that the Osaka mutation impairs the APP function necessary for the formation and maintenance of GABAergic synapses. If this were the case, homozygous OSK-KI mice would show deficient GABAergic transmission in the dentate gyrus, which presumably leads to abnormal synaptic activation and resultant memory impairment. Our data appear to support this theory: 4-month-old homozygotes displayed decreased levels of dentate GABAergic neurons, abnormal LTP induction, and impaired memory. This GABAergic depletion was not likely caused by Aβ oligomers, because Aβ accumulation was first detected at 8 months and because only GABAergic, but not glutamatergic, neurons were affected at 4 months. Furthermore, only dentate, but not entorhinal, GABAergic neurons were significantly decreased, despite that the both regions accumulated Aβ oligomers. We also showed that the memory impairment in homozygotes could be rescued by oral administration of diazepam, an allosteric modulator of GABA_A_ receptor to promote GABA binding and thereby enhance GABAergic inhibitory input. This finding further supports our theory that GABAergic depletion is a cause of memory disturbance.

**Fig. 8:**
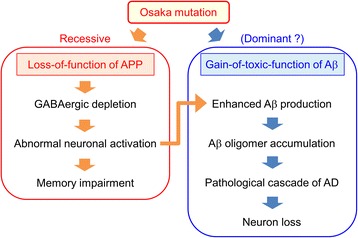
A proposed mechanism for the recessive heredity of the Osaka mutation. The Osaka mutation has dual effects; a loss-of-function of APP leading to GABAergic depletion and gain-of-toxic-function of Aβ to accelerate its oligomerization. While the former effect accounts for the recessive inheritance of this mutation, the latter seems to be a dominant effect to cause dementia even in heterozygotes. But practically, Aβ oligomer accumulation is not detected until GABAergic depletion proceeds. This is probably because Aβ production is negatively regulated by GABAergic inhibitory input. Once Aβ oligomers accumulate, it triggers pathological cascade of AD, including abnormal tau phosphorylation, synapse loss, glial activation, and eventual neuron loss

Homozygous OSK-KI mice, which express only mutant mouse APP, exhibited a marked accumulation of Aβ oligomers at 8 months similarly to APP_OSK_ mice that overexpress mutant human APP. This finding indicates that the toxic effect of the Osaka mutation on Aβ oligomerization is strong enough to be displayed not only in the human but also in the mouse Aβ sequence. We confirmed this conclusion in transfected cells, in which Osaka-mutant mouse Aβ formed oligomers more abundantly than wild-type, similarly to Osaka-mutant human Aβ. Nevertheless, the phenotype was detected only in homozygotes. While many other APP mutations that affect Aβ aggregation are dominant, why does the Osaka mutation show recessive inheritance? We noticed that Aβ oligomer accumulation was detected only after the onset of GABAergic depletion. This order of appearance might indicate some causal relationship between these two events. Aβ production has been shown to rely on neuronal activity [8, 17], and GABA is an inhibitory neurotransmitter. Thus, it is likely that Aβ production is negatively regulated by GABAergic transmission. If so, GABAergic deficiency would result in an accelerated Aβ production and accumulation via abnormal synaptic activation. This story is plausible because diazepam treatment to homozygotes from 6 months prevented Aβ oligomer accumulation at 8 months. This explains why the Osaka mutation shows recessive inheritance in spite of its gain-of-toxic-function: Accumuation of Aβ oligomers would not occur until Aβ production is increased by GABAergic depletion (Fig. 8). Intraneuronal accumulation of Aβ has been shown to be an early event in both human patients and mouse models of AD [6, 9, 15, 16, 23, 31]. Once Aβ oligomers accumulate, it triggers other neuropathologies of AD, such as abnormal tau phosphorylation, synapse loss, glial activation, and eventual neuron loss, as previously demonstrated in APP_OSK_ mice [26].

Regarding the recessive heredity of the Osaka mutation, some other possibilities are also considered. First, we cannot exclude the possibility that the recessive appearance of the Osaka mutation is due to its incomplete penetrance or variable expressivity. However, we have not observed such symptoms in our OSK-KI mice except for synapse loss in 24-month-old heterozygotes. Second, it may be that the presence of wild-type Aβ interferes with the oligomerization of mutant Aβ, like the aforementioned recessive APP mutation A673V [5]. This is unlikely, however, because APP_OSK_ mice that express both mutant and wild-type Aβ at similar levels showed Aβ oligomer accumulation [26]. Third, whether Aβ oligomers accumulate in the brain may simply depend on the concentration of mutant Aβ. Homozygotes express a sufficient amount of mutant Aβ, whereas heterozygotes produce only a half amount that in homozygotes, not reaching pathogenic levels. We assume that even in homozygotes, the amount of mutant Aβ is insufficient and GABAergic depletion is necessary for Aβ oligomer accumulation, as described above.

GABAergic dysfunction may underlie the pathogenesis of AD, not only in the Osaka mutation but also in other familial and sporadic cases. For example, it has been reported that the levels of GABA are reduced in the posterior cingulated cortex of amnestic mild cognitive impairment independently of amyloid deposition [19] and in the parietal cortex of patients with AD [1]. Neuronal hyperactivity has also been observed in the presymptomatic stages of both sporadic and familial AD [21]. Furthermore, two major Aβ-degrading enzymes, endothelin-converting enzyme-2 and neprilysin, were shown to be enriched in GABAergic interneurons in the hippocampus and neocortex [18], implying that GABAergic neuron loss results in lowered degradation and subsequent accumulation of Aβ. In this regard, it is noteworthy that previous use of benzodiazepine has been shown to be associated with lower cortical Aβ levels in non-demented elderly control subjects [4]. These findings collectively implicate that pharmacological treatments to compensate GABAergic deficiency might have therapeutic potential in early stages of AD [12].

## Conclusions

In summary, we elucidated here that the Osaka mutation has dual effects: it causes a loss-of-function of APP and gain-of-toxic-function of Aβ, though the latter seems to come out only after the former causes GABAergic depletion. To our knowledge, the present OSK-KI mice is the first mouse model to replicate the hereditary form of recessive familial AD, though the phenotypes are not yet validated in human cases. Furthermore, the present study demonstrates for the first time that mutation-induced loss of APP function could be a cause of recessive hereditary dementia.
